# Update on the pathogenesis of Scleroderma: focus on circulating progenitor cells

**DOI:** 10.12688/f1000research.7986.1

**Published:** 2016-04-22

**Authors:** Alexandra Maria Giovanna Brunasso, Cesare Massone

**Affiliations:** 1 Department of Dermatology, Medical University of Graz, Auenbruggerplatz 8, A-8036 Graz, Austria; 2 Department of Dermatology, Galliera Hospital, Genoa, Italy

**Keywords:** fibrosis, CD14+ cells, pluripotent stem cells, monocyte-derived multipotential cells, early endothelial progenitor cells, monocytic pro-angiogenic hematopoietic cells, fibroblast-like cells, fibrocytes

## Abstract

In systemic sclerosis (SSc), the development of fibrosis seems to be a consequence of the initial ischemic process related to an endothelial injury. The initial trigger event in SSc is still unknown, but circulating progenitor cells (CPCs) might play a key role. Such cells have the ability to traffic into injury sites, exhibiting inflammatory features of macrophages, tissue remodeling properties of fibroblasts, and vasculogenesis functions of endothelial cells. The different subsets of CPCs described thus far in SSc arise from a pool of circulating monocyte precursors (CD14 ^+^ cells) and probably correspond to a different degree of differentiation of a single cell of origin. Several subsets of CPCs have been described in patients with SSc, all have a monocytic origin but may or may not express CD14, and all of these cells have the ability to give origin to endothelial cells, or collagen (Col)-producing cells, or both. We were able to identify six subsets of CPCs: pluripotent stem cells (CD14 ^+^, CD45 ^+^, and CD34 ^+^), monocyte-derived multipotential cells (MOMCs) or monocyte-derived mesenchymal progenitors (CD14 ^+^, CD45 ^+^, CD34 ^+^, Col I ^+^, CD11b ^+^, CD68 ^+^, CD105 ^+^, and VEGFR1 ^+^), early endothelial progenitor cells (EPCs) or monocytic pro-angiogenic hematopoietic cells or circulating hematopoietic cells (CD14 ^+^, CD45 ^+^, CD34 ^low/−^, VEGFR2 ^+/−^, CXCR4 ^+^, c-kit ^+^, and DC117 ^+^), late EPCs (CD14 ^−^, CD133 ^+^, VEGFR2 ^+^, CD144 ^+^ [VE-cadherin ^+^], and CD146 ^+^), fibroblast-like cells (FLCs)/circulating Col-producing monocytes (CD14 ^+^, CD45 ^+^, CD34 ^+/−^, and Col I ^+^), and fibrocytes (CD14 ^−^, CD45 ^+^, CD34 ^+^, Col I ^+^, and CXCR4 ^+^). It has been demonstrated that circulating CD14 ^+^ monocytes with an activated phenotype are increased in patients with SSc when compared with normal subjects. CD14 ^+^, CD34 ^+^, and Col I ^+^ spindle-shaped cells have been found in increased numbers in lungs of SSc patients with interstitial lung disease. Elevated blood amounts of early EPCs have been found in patients with SSc by different groups of researchers and such levels correlate directly with the interstitial lung involvement. The prevalence of hematopoietic markers expressed by CPCs that migrate from blood into injury sites in SSc differs and changes according to the degree of differentiation. CXCR4 is the most commonly expressed marker, followed by CD34 and CD45 at an end stage of differentiation. Such difference also indicates a continuous process of cell differentiation that might relate to the SSc clinical phenotype (degree of fibrosis and vascular involvement). A deeper understanding of the role of each subtype of CPCs in the development of the disease will help us to better classify patients in order to offer them targeted approaches in the future.

## Introduction

Systemic sclerosis (SSc) is a chronic, complex, and not yet completely understood autoimmune disease characterized by the presence of immunological events, the onset of fibrosis, and the development of vascular alterations ^1^. The development of fibrosis seems to be a consequence of the initial ischemic process related to an endothelial injury ^2^. In the early disease phase (first year from diagnosis), there is an increase of pro-angiogenic factors—vascular endothelial growth factor (VEGF), platelet-derived growth factor, and stromal-derived growth factor-1—in response to the vascular damage that correlates to the nailfold capillaroscopy changes observed by clinicians (giant capillaries and new vessel formation) ^3^. But in the late phase of SSc, the anti-angiogenic response and the fibrosis seem to dominate over the initial pro-angiogenic phase ^4^. The initial trigger event in SSc is still unknown, but circulating progenitor cells (CPCs) might play a key role ^4^.

Since 1994, when Bucala *et al.* first described these fibrocytes as circulating leukocytes able to produce collagen (Col), different types of progenitor cells have been described as key players in different entities, such as pulmonary artery hypertension (PAH), asthma, interstitial lung disease (ILD), idiopathic pulmonary fibrosis, and sclerosing diseases (cirrhosis, atherosclerosis, SSc, chronic kidney disease, and so on), and as initially described in wound healing. Such cells have the ability to traffic into injury sites, exhibiting both inflammatory features of macrophages and tissue remodeling properties of fibroblasts ^5^. Interestingly, some of the CPCs are also able to differentiate into endothelial cells playing a role in the vasculogenesis process ^5^. In this review, we will summarize the existing evidence regarding the role of CPCs in SSc.

## Analysis of the recent literature

### Progenitor cells in systemic sclerosis

Since 1994, different subtypes of CPCs have been described as quantitatively or functionally altered in patients with SSc ( Table 1) ^6– 19^. The different subtypes of CPCs described thus far in SSc arise from a pool of circulating monocyte precursors (CD14 ^+^ cells) and probably correspond to a different degree of differentiation from a single cell of origin. The pluripotent stem cells (PSCs) seem to correspond to a very early degree of differentiation. The monocyte-derived mesenchymal progenitors (MOMCs), also known as monocyte-derived mesenchymal progenitors, are multipotent cells with a spindle-shaped morphology and a unique phenotype (CD14 ^+^, CD45 ^+^, CD34 ^+^, and type I Col ^+^) that might correspond to the PSCs described by Zhao *et al.* ^6^, but with the ability to produce Col (a further degree of differentiation). MOMCs show mixed morphologic and phenotypic features of phagocytes, mesenchymal cells, and endothelial cells ^[Bibr ref-2], 4, 8^. It has been demonstrated that circulating CD14 ^+^ monocytes with an activated phenotype (CD68 ^+^, CD204 ^+^, and Singlec-1 ^+^) are increased in patients with SSc when compared with normal subjects ^12– 14, 16^. CD14 ^+^, CD34 ^+^, and Col I ^+^ spindle-shaped cells (compatible with MOMCs) have been found in increased numbers in the lungs of SSc patients with ILD ^15, 16^. In 2004, Postlethwaite *et al.* reported an elevated number of spindle-shaped cells with a new phenotype (CD14 ^+^, CD45 ^+^, CD34 ^−^, and Col I ^+^) in patients with SSc after culturing peripheral blood monocyte cells (PBMCs) with type I Col. They called these particular cells fibroblast-like cells (FLCs). They differ from the already-known PSCs and MOMCs by the absence of CD34 expression and from fibrocytes by the presence of CD14 and the absence of CD34 as surface markers ^11^. They also suggested that the increased outgrowth of FLCs from patients with SSc may be a marker of diffuse disease and pulmonary fibrosis ^11^.

Other subtypes of CPCs have been related to the vascular alterations seen in patients with SSc, as the circulating endothelial precursor cells (EPCs) that seem to contribute to the initial phase of SSc. In 2009, the European League Against Rheumatism (EULAR) Scleroderma Trials and Research group provided recommendations for standardization for future research in EPCs ^10^. The consensus panel agreed to classify EPCs in two groups:

1. *Early EPCs*, characterized by the positive expression of CD14, CD45, the low expression of CD34, and the variable expression of VEGFR2 (+/−), have also been described as monocytic pro-angiogenic hematopoietic cells by Yamaguchi *et al.* ^2^ and as circulating hemotopoietic progenitor cells (CD45 ^+^, CXCR4 ^+^, c-kit ^+^, and CD117 ^+^) by Campioni *et al.* ^9^. Early EPCs have the ability to differentiate into endothelial cells, fibroblasts, smooth muscle cells, and pericytes ^2^. Elevated blood levels of early EPCs have been found in patients with SSc by different groups of researchers and such levels correlate directly with interstitial lung involvement ^2, 9^. In a pro-fibrotic environment—elevated levels of both endothelin-1 (ET-1) and transforming growth factor-beta (TGF-β)—early EPCs differentiate mainly into fibroblasts and promote fibrosis ^2^.

2. *Late EPCs* are a population of bone marrow-derived cells that are characterized by the phenotype CD14 ^−^, CD34 ^+^, CD133 ^+^, and VEGFR2 ^+^, and that are able to differentiate into mature endothelial cells and participate in vasculogenesis. In patients affected by SSc, late EPCs are able to differentiate into myofibroblasts and play a role in the development of fibrosis ^20^. The number of circulating late EPCs is inversely proportional to the SSc disease duration: in the early phase such cells are increased and at late stages they are decreased, as confirmed by different authors ^4, 9, 10, 20^. Lower levels of late EPCs particularly are found in patients with past or current digital ulcers and PAH ^20^. Such cells not only decrease in a late phase of SSc but are also functionally impaired and resistant to *in vitro* maturation treatments, suggesting a defect in the vasculogenesis process (the failure of new blood vessel formation because of a failure in recruitment and *in situ* differentiation of late EPCs) ^4^. A plausible explanation for the decrease of late EPCs during the late phase of SSc regards the recruitment into injured tissues of such cells, decreasing the circulating numbers ^20^.

**Table 1.** CPCs described in SSc.

CPCs subtypes	Author/Date	Cellular markers	Alteration in SSc (increase/ decrease)	Differentiation capability
Pluripotent Stem Cells (PSC)	Zhao *et al.*, 2003 ^6^	CD14+, CD45+, CD34+	Increased	Macrophages, T-lymphocytes, epithelial, neuronal cells, hepatocytes and endothelial cells. Fibroblasts (unknown)
Monocyte-derived multipotential cells (MOMCs) or monocyte-derived mesenchymal progenitors (MOMPs)	Kuwana *et al.*, 2003, Seta *et al.*, 2010, Yamaguchi *et al.*, 2013 ^2, 6– 8^	CD14+, CD45+, CD34+, Col I+, CD11b+, CD68+, CD-105+, VEGFR1+	Increased	Endothelial cells, fibroblasts, hematopoietic and monocyte lineage, phagocytes, mesenchymal cells
Early endothelial progenitor cells (EPCs) or monocytic pro-angiogenic hematopoietic cells (PHC) or circulating hematopoietic cells	Kuwana *et al.*, 2004, Campioni *et al.*, 2008, Distler *et al.*, 2009, Yamaguchi *et al.*, 2013, ^2, 4, 9, 10^	CD14+, CD45+, CD34low/-, VEGFR2+/-, CXCR4+, c-kit+, DC117+	Increased and directly correlate with interstitial lung involvement	Smooth muscle cells, pericytes, endothelial cells, fibroblasts
Late-EPCs:	Kuwana *et al.*, 2004, Campioni *et al.*, 2008, Distler *et al.*, 2009 ^4, 9, 10^	CD14-, CD133+, VEGFR2+, CD144+ (VE- cadherin)+, CD146+	Increase in early phase or with severe vascular involvement (PAH, SRC, Ray) and decrease in late phase or fibrosis. Not only quantitative alterations but also in SSc patients functional impairment (defective vasculogenesis)	Myofibroblasts and endothelial cells
Fibroblasts like cells (FLC)/circulating collagen producing monocytes (CPM):	Postlethwaite *et al.*, 2004, York *et al.*, 2007, Higashi-Kuwata *et al.*, 2010, Mathai *et al.*, 2010, Tourkina *et al.*, 2011, Binai *et al.*, 2012 ^10– 16^	CD14+, CD45+, CD34+/-, Col I+	Increased not only in circulation but also in lungs of SSc patients with interstitial lung disease.	Myofibroblasts and endothelial cells
Fibrocytes:	Schmidt *et al.*, 2003, Quan *et al.*, 2004, Strieter *et al.*, 2009 ^5, 17– 19^	CD14-, CD45+, CD34+, Col I+, CXCR4+	Increased	Fibroblasts, myofibroblasts

### The role of circulating progenitor cells in systemic sclerosis

The importance of CPCs relies on the capacity of such cells to migrate into SSc injury tissues (mediated by CXCR4 /CXCL12 interaction), to differentiate into both endothelial cells and fibroblasts, to cause defective vasculogenesis or fibrosis (or both), and to have immunomodulatory effects ^19^. The prevalence of hematopoietic markers expressed by CPCs that migrate from blood into injury sites in SSc differs and changes according to the degree of differentiation. CXCR4 is the most commonly expressed marker, followed by CD34 and CD45 at the end stage of differentiation ^15^. Such difference also indicates a continuous process of cell differentiation that might relate to the SSc clinical phenotype (degree of fibrosis and vascular involvement).

### Circulating progenitor cells and fibrosis

In patients with SSc, the fraction of CD14 ^+^ monocytes in circulation is higher than the CD14 ^−^ monocytes and a greater portion of circulating monocytes express Col I, suggesting that SSc monocyte preparations may contain a significant number of Col-producing cells that are partially differentiated into different subtypes of CPCs (MOMCs, FLCs, EPCs, and fibrocytes) ^14^. Such circulating Col-producing cells have an increased migration capacity into injury sites because of the overexpression of CXCR4 and the deficiency in caveolin-1. CPCs that have finished their differentiation process generate fibrocytes that produce Col, extracellular matrix and cause fibrosis at injury sites (skin, lung, kidneys, and so on) ^15^. Interestingly, it has been described that African-Americans may be predisposed to lung fibrosis and SSc because of low baseline caveolin-1 levels in their monocytes, potentially affecting signaling, migration, and fibrocyte differentiation ^21^. The finding that CD14 ^+^/Col I ^+^ monocytes are present in the lung tissue of patients with SSc-ILD and not in healthy donors supports this hypothesis ^15^. It is interesting that, in a pro-fibrotic environment (elevated levels of ET-1 and TGF-β), early EPCs that normally give rise to endothelial cells can also differentiate into FLCs and promote fibrosis ^2^. Fibrosis occurs after the activation of tissue-resident fibroblasts and their transdifferentiation into myofibroblasts, but is also due to differentiation of bone marrow-derived CPCs and transition of endothelial epithelial cells, pericytes, and adipocytes into activated mesenchymal cells ^1, 22, 23^. Fibrocytes (CD14 ^−^, CD34 ^+^, CD45 ^+^, CXCR4 ^+^, CCR3, and Col I ^+^), defined as FLCs that differentiate from a different pool of bone marrow-derived monocytic CD14 ^+^ progenitor cells, are involved in both ischemic and fibrotic processes in SSc ^8, 19, 22, 23^. It is worth noting that CD14 ^+^ circulating monocytes in the presence of T cells give rise to fibrocytes (CD14 ^−^ cells) ^22^. Fibrocytes cultured with TGF-β or ET-1 downregulate CD34 and upregulate alpha-smooth muscle actin (α-SMA) expression and differentiate into myofibroblasts ^17^. Fibrocytes are considered to be mesenchymal cells that arise from a pool of circulating monocyte precursors ^24^. The number of circulating fibrocytes in patients with idiopathic pulmonary fibrosis directly correlates with exacerbations of the disease, and patients with fibrocytes more than 5% of total circulating blood leukocytes had a worse prognosis than patients with levels under this cut-off ^19^.

### Quantification of circulating progenitor cells in systemic sclerosis

Both late EPCs and FLCs have been found to be significantly increased not only in blood of patients with SSc but also at injury sites (lungs with ILD-SSc) ^20^. Late EPCs contribute to new vessel formation and vascular repair via secretion angiogenic factors under normal conditions, a mechanism that is disrupted in patients with SSc ^20^. FLCs are key effectors of fibrosis ^11^. The number of circulating late EPCs is inversely proportional to the disease duration ^10, 21^. Lower levels of late EPCs are found in the late phase of SSc, in patients with diffuse fibrosis, and particularly in patients with past or current digital ulcers and PAH ^21^. Late EPCs not only decrease in a late phase of SSc but also are functionally impaired and resistant to *in vitro* maturation treatment, suggesting a defective vasculogenesis (the failure in new blood vessel formation because of a failure in recruitment and *in situ* differentiation of late EPCs) ^4^. The total number of circulating CD45 ^+^/pro-Col-1α cells (that might correspond to FLCs and fibrocytes) has been reported to be 0.34 ± 0.12 × 10 ^6^ cells/ml, and the percentage of such cells between PBMCs to be between 1.34 ± 0.25 (ILD-SSc) and 2.5% (SSc) ^10, 15^. CD14 ^+^, CD11b ^+^, and Col I ^+^ cells have been reported to be 1.5% of PBMCs in patients with SSc, versus 0.95% in healthy donors ( *P* <0.05) ^14^. EPC frequency in peripheral blood is quite low: 0.01% to 0.0001% of PBMCs ^20^.

## Conclusions and future perspective of circulating progenitor cells in systemic sclerosis

In the past 15 years, a pool of circulating monocyte precursors has been found to be altered both quantitatively and qualitatively in different sclerosing conditions, including SSc. Such alterations have been partially related to the subtype of SSc (diffuse versus limited forms), the duration of the SSc, and the prominent clinical manifestations (vascular involvement versus fibrosis). The exact role of each subtype of CPCs needs to be further defined. A deeper understanding of the role of each subtype of CPCs in the development of the disease will help us to better classify patients in order to offer them personalized therapeutic approaches in the future ^25– 28^. It might also open a door regarding the modulation/regulation of the differentiation of CPCs in order to avoid a pro-fibrotic phenotype and to reverse the altered vascular phenotype of such cells. Autologous hematopoietic stem cell transplantation (HSTC) seems to reintroduce immunological tolerance in patients with SSc, and we hypothesize that such tolerance might be due to the regulation of CPC differentiation. It has been demonstrated that after HSTC there is an improvement of vasculopathy, modified Rodnan skin score, and lung function in patients with SSc ^26^.

This review had the intention to summarize the available data regarding CPCs in SSc; considering the fact that several studies have been conducted with single CPC subsets, we herein intended to describe the whole spectrum of CPCs in SSc described thus far and their roles in the pathogenesis of SSc. During this review process, we faced several difficulties: the same cell has been studied by different research groups and named differently, several subsets of CPCs that might correspond to the same cell have been described, there are no studies available that compare different subsets of CPCs in the same patients, and there is no direct correlation in the literature between different CPC subsets and clinical manifestations of SSc.

## References

[1] Wei J Bhattacharyya S Tourtellotte WG: Fibrosis in systemic sclerosis: emerging concepts and implications for targeted therapy. *Autoimmun Rev.* 2011;10(5):267–275. 10.1016/j.autrev.2010.09.015 20863909 PMC3998379

[ref-2] Yamaguchi Y Kuwana M: Proangiogenic hematopoietic cells of monocytic origin: roles in vascular regeneration and pathogenic processes of systemic sclerosis. *Histol Histopathol.* 2013;28(2):175–183. 10.14670/HH-28.175 23275301

[3] Cutolo M Sulli A Smith V: Assessing microvascular changes in systemic sclerosis diagnosis and management. *Nat Rev Rheumatol.* 2010;6(10):578–587. 10.1038/nrrheum.2010.104 20703220

[4] Kuwana M Okazaki Y Yasuoka H: Defective vasculogenesis in systemic sclerosis. *Lancet.* 2004;364(9434):603–610. 10.1016/S0140-6736(04)16853-0 15313361

[5] Bucala R Spiegel LA Chesney J: Circulating fibrocytes define a new leukocyte subpopulation that mediates tissue repair. *Mol Med.* 1994;1(1):71–81. 8790603 PMC2229929

[6] Zhao Y Glesne D Huberman E: A human peripheral blood monocyte-derived subset acts as pluripotent stem cells. *Proc Natl Acad Sci U S A.* 2003;100(5):2426–2431. 10.1073/pnas.0536882100 12606720 PMC151357

[7] Kuwana M Okazaki Y Kodama H: Human circulating CD14 ^+^ monocytes as a source of progenitors that exhibit mesenchymal cell differentiation. *J Leukoc Biol.* 2003;74(5):833–845. 10.1189/jlb.0403170 12960274

[8] Seta N Kuwana M: Derivation of multipotent progenitors from human circulating CD14 ^+^ monocytes. *Exp Hematol.* 2010;38(7):557–563. 10.1016/j.exphem.2010.03.015 20362030

[9] Campioni D Lo Monaco A Lanza F: CXCR4 ^pos^ circulating progenitor cells coexpressing monocytic and endothelial markers correlating with fibrotic clinical features are present in the peripheral blood of patients affected by systemic sclerosis. *Haematologica.* 2008;93(8):1233–1237. 10.3324/haematol.12526 18556411

[10] Distler JHW Allanore Y Avouac J: EULAR Scleroderma Trials and Research group statement and recommendations on endothelial precursor cells. *Ann Rheum Dis.* 2009;68(2):163–168. 10.1136/ard.2008.091918 18653485

[11] Postlethwaite AE Shigemitsu H Kanangat S: Cellular origins of fibroblasts: possible implications for organ fibrosis in systemic sclerosis. *Curr Opin Rheumatol.* 2004;16(6):733–738. 10.1097/01.bor.0000139310.77347.9c 15577612

[12] York MR Nagai T Mangini AJ: A macrophage marker, Siglec-1, is increased on circulating monocytes in patients with systemic sclerosis and induced by type I interferons and toll-like receptor agonists. *Arthritis Rheum.* 2007;56(3):1010–1020. 10.1002/art.22382 17328080

[13] Higashi-Kuwata N Jinnin M Makino T: Characterization of monocyte/macrophage subsets in the skin and peripheral blood derived from patients with systemic sclerosis. *Arthritis Res Ther.* 2010;12(4):R128. 10.1186/ar3066 20602758 PMC2945018

[14] Mathai SK Gulati M Peng X: Circulating monocytes from systemic sclerosis patients with interstitial lung disease show an enhanced profibrotic phenotype. *Lab Invest.* 2010;90(6):812–823. 10.1038/labinvest.2010.73 20404807 PMC3682419

[15] Tourkina E Bonner M Oates J: Altered monocyte and fibrocyte phenotype and function in scleroderma interstitial lung disease: reversal by caveolin-1 scaffolding domain peptide. *Fibrogenesis Tissue Repair.* 2011;4(1):15. 10.1186/1755-1536-4-15 21722364 PMC3155832

[16] Binai N O'Reilly S Griffiths B: Differentiation potential of CD14+ monocytes into myofibroblasts in patients with systemic sclerosis. *PloS One.* 2012;7(3):e33508. 10.1371/journal.pone.0033508 22432031 PMC3303833

[17] Schmidt M Sun G Stacey MA: Identification of circulating fibrocytes as precursors of bronchial myofibroblasts in asthma. *J Immunol.* 2003;171(1):380–389. 10.4049/jimmunol.171.1.380 12817021

[18] Quan TE Cowper S Wu S: Circulating fibrocytes: collagen-secreting cells of the peripheral blood. *Int J Biochem Cell Biol.* 2004;36(4):598–606. 10.1016/j.biocel.2003.10.005 15010326

[19] Strieter RM Keeley EC Hughes MA: The role of circulating mesenchymal progenitor cells (fibrocytes) in the pathogenesis of pulmonary fibrosis. *J Leukoc Biol.* 2009;86(5):1111–1118. 10.1189/jlb.0309132 19581373 PMC2774878

[20] Avouac J Juin F Wipff J: Circulating endothelial progenitor cells in systemic sclerosis: association with disease severity. *Ann Rheum Dis.* 2008;67(10):1455–1460. 10.1136/ard.2007.082131 18174219

[21] Reese C Perry B Heywood J: Caveolin-1 deficiency may predispose African Americans to systemic sclerosis-related interstitial lung disease. *Arthritis Rheumatol.* 2014;66(7):1909–1919. 10.1002/art.38572 24578173 PMC4158912

[22] Struyf S Burdick MD Proost P: Platelets release CXCL4L1, a nonallelic variant of the chemokine platelet factor-4/CXCL4 and potent inhibitor of angiogenesis. *Circ Res.* 2004;95(9):855–857. 10.1161/01.RES.0000146674.38319.07 15459074

[23] Zaldivar MM Pauels K von Hundelshausen P: CXC chemokine ligand 4 (Cxcl4) is a platelet-derived mediator of experimental liver fibrosis. *Hepatology.* 2010;51(4):1345–1353. 10.1002/hep.23435 20162727

[24] Bucala R: Fibrocytes at 20 Years. *Mol Med.* 2015;21(Suppl 1):S3–5. 26605645 10.2119/molmed.2015.00043 PMC4661049

[25] Struyf S Salogni L Burdick MD: Angiostatic and chemotactic activities of the CXC chemokine CXCL4L1 (platelet factor-4 variant) are mediated by CXCR3. *Blood.* 2011;117(2):480–488. 10.1182/blood-2009-11-253591 20980681 PMC3031477

[26] Cutolo M Sulli A Pizzorni C: Systemic sclerosis: markers and targeted treatments. *Acta Reumatol Port.* 2016. 27115104

[27] van Bon L Affandi AJ Broen J: Proteome-wide analysis and CXCL4 as a biomarker in systemic sclerosis. *N Engl J Med.* 2014;370(5):433–443. 10.1056/NEJMoa1114576 24350901 PMC4040466

[28] Vandercappellen J van Damme J Struyf S: The role of the CXC chemokines platelet factor-4 (CXCL4/PF-4) and its variant (CXCL4L1/PF-4var) in inflammation, angiogenesis and cancer. *Cytokine Growth Factor Rev.* 2011;22(1):1–18. 10.1016/j.cytogfr.2010.10.011 21111666

